# The American Medical Association

**Published:** 1883-07

**Authors:** 


### The American Medical Association.

The thirty-fourth annual meeting of this association, held in Cleveland June 5th to 8th, inclusive, was very largely attended, eleven hundred delegates and permanent members having signed the register.

It is a question how many "strained their consciences" or considered it right to simply ignore the clause requiring entire agreement with the old Code, which had been interpolated by the "Judicial Counsel" and which is referred to by a valued exchange as "a dexterity by which all ethical rebels were strangled at the door so that none succeeded in crossing the sacred æsculapian threshold, and the shades of Hippocrates suffered no dishonor." The writer is not a supporter of the "New Code," but we are always on the side of friendly and free discussion, and we must decidedly protest at the "dexterity" by which this was strangled. It is an impertinence to the members of the association to assume that they can never change or modify their views, and that having, perhaps years ago, honestly pledged themselves to the present Code, they are not at liberty to as honestly change their minds and by all proper means to advocate their views in the association. The action of the "Judicial Counsel" virtually deprived every permanent member, whose views had so changed, of his rights and privileges in the association, and it is a discredit to the majority that such an outrage upon the rights of a few should have been permitted. Such action gives to the advocates of the New Code the best apology for the unfortunate position assumed by the New York State Society, and furthermore, will tend more powerfully than argument to draw fair-minded men to their side.

The election of Dr. Austin Flint to the presidency will be a gratification to all medical men, but can add little to the honors with which he is already crowned. In Europe, as here, he is recognized as the foremost medical man in America. One of the most important matters accomplished at the meeting was that relating to the journalizing of the transactions. Under the able and experienced editorship of Dr. N. S. Davis, we can confidently predict that the Journal will be a fit representative of the American Medical Association. The first number is expected to appear early in July, and as it will undoubtedly reach most of our subscribers, we have omitted our usual report of the proceedings of the association, which otherwise we would have felt it necessary to publish.

